# Genome-Wide Mutagenesis Links Multiple Metabolic Pathways with Actinorhodin Production in Streptomyces coelicolor

**DOI:** 10.1128/AEM.03005-18

**Published:** 2019-03-22

**Authors:** Zhong Xu, Yuanyuan Li, Yemin Wang, Zixin Deng, Meifeng Tao

**Affiliations:** a Institute of Business Scientific, Henan Academy of Sciences, Zhengzhou, Henan, China; b State Key Laboratory of Microbial Metabolism, Joint International Research Laboratory of Metabolic & Developmental Sciences, and School of Life Sciences and Biotechnology, Shanghai Jiao Tong University, Shanghai, China; North Carolina State University

**Keywords:** genome wide, *Streptomyces coelicolor*, actinorhodin, antibiotic biosynthesis, transposition mutagenesis

## Abstract

*Streptomyces* species are important antibiotic-producing organisms that tightly regulate their antibiotic production. Actinorhodin is a typical antibiotic produced by the model actinomycete Streptomyces coelicolor. To discover the regulators of actinorhodin production, we constructed a library of 50,000 independent mutants with hyperactive Tn*5* transposase-based transposition systems. Five hundred fifty-one genes were found to influence actinorhodin production in 988 individual mutants. Genetic complementation suggested that most of the insertions (76%) were responsible for the changes in antibiotic production. Genes involved in diverse cellular processes such as amino acid biosynthesis, carbohydrate metabolism, cell wall homeostasis, and DNA metabolism affected actinorhodin production. Genome-wide mutagenesis can identify novel genes and pathways that impact antibiotic levels, potentially aiding in engineering strains to optimize the production of antibiotics in *Streptomyces*.

**IMPORTANCE** Previous studies have shown that various genes can influence antibiotic production in *Streptomyces* and that intercommunication between regulators can complicate antibiotic production. Therefore, to gain a better understanding of antibiotic regulation, a genome-wide perspective on genes that influence antibiotic production was needed. We searched for genes that affected production of the antibiotic actinorhodin using a genome-wide gene disruption system. We identified 551 genes that altered actinorhodin levels, and more than half of these genes were newly identified effectors. Some of these genes may be candidates for engineering *Streptomyces* strains to improve antibiotic production levels.

## Abstract (precis)

Previous studies have shown that various genes can influence antibiotic production in *Streptomyces* and that intercommunication between regulators can complicate antibiotic production. Therefore, to gain a better understanding of antibiotic regulation, a genome-wide perspective on genes that influence antibiotic production was needed. We searched for genes that affected production of the antibiotic actinorhodin using a genome-wide gene disruption system. We identified 551 genes that altered actinorhodin levels, and more than half of these genes were newly identified effectors. Some of these genes may be candidates for engineering *Streptomyces* strains to improve antibiotic production levels.

## INTRODUCTION

*Streptomyces* species are important antibiotic producers because of their ability to generate many compounds with antibiotic, immunosuppressive, anticancer, and antihelminthic activities (1). With the development of genome sequencing technologies, a growing number of *Streptomyces* genome sequences are now available (2). Genome mining results have shown that the average number of secondary metabolite gene clusters in each *Streptomyces* genome is approximately 20 (3–5), but only one-tenth of these metabolites are detectable during fermentation, as the products of most clusters are too low to be detected. Various methods have been reported to enhance antibiotic production and/or activate the biosynthesis of cryptic antibiotics, including heterologous expression (6, 7), metal and nutrient stress (8–11), the addition of small molecules, such as ARC2 (12), physical interaction between different microorganisms (13), and increasing the copy number of antibiotic biosynthesis genes (14). Therefore, the potential for the discovery of novel drugs in *Streptomyces* is enormous, and the identification of factors that regulate antibiotic production in this genus is important for maximizing the value of these novel drugs.

Streptomyces coelicolor A3(2) was the first *Streptomyces* strain to have its genome completely sequenced and is the best-studied *Streptomyces* strain (3). S. coelicolor produces two pigmented model antibiotics, the red-pigmented antibiotic undecylprodigiosin (RED) and the blue-pigmented antibiotic actinorhodin (ACT) (3). ACT is a member of the benzoisochromanequinone class of polyketide antibiotics and is synthesized by a typical type II polyketide synthase (PKS) gene cluster; the functions of most enzymes encoded by this gene cluster have been elucidated (15–18). ACT is an ideal model to identify regulators of antibiotic production. ACT production is modulated by transcriptional regulators such as AfsR, WblA, DasR, AbaA, and XdhR (10, 19–22) and by other proteins such as OrnA, CmdA to CmdE, and SarA (23–25).

Many studies have been conducted to elucidate the regulation of antibiotic production in *Streptomyces*, predominantly in S. coelicolor, and such studies have used DNA microarrays to reveal gene expression patterns during metabolic switching (26, 27), allowed the construction of genome-wide metabolic models (28–30), and identified transcriptional regulators required for antibiotic production (31–35). These studies have shown that the expression of antibiotic cluster genes is tightly regulated and that substrate and energy supplies are crucial for antibiotic production. To genetically manipulate antibiotic production, groundwork studies are first needed to identify genes that influence antibiotic production in a genome-wide manner. To identify regulators of ACT production, we searched for regulatory genes involved in ACT production in the model S. coelicolor strain M145 using Tn*5*-based *in vivo* transposon mutagenesis (36). We identified 551 genes that influenced ACT production, and more than half of these genes are newly identified effectors. The results of gene complementation confirmed that most of these genes are ACT modulators. Some of these genes may play important roles for strain improvement.

## RESULTS

### Genome-wide transposon screening for genes that affect ACT production.

**FIG 1:**
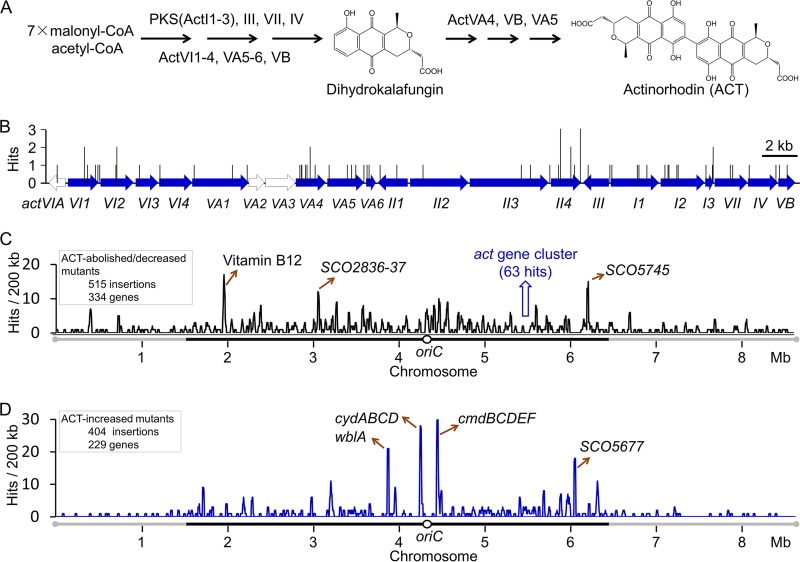
Distribution of mini-Tn*5* insertions affecting ACT production along the S. coelicolor chromosome. (A) The ACT biosynthetic pathway. (B) Mini-Tn*5* insertions in the *act* locus. (C) Distribution of insertions outside the *act* gene cluster that abolished/decreased ACT production. (D) Distribution of insertions that led to increased ACT production. Sliding window with a step size of 20 kb. Genes in peaks with more than ten hits are indicated. The segments below the plots in panels C and D indicate the core (black) and arm (gray) regions of the chromosome. *oriC*, origin of replication.

To identify genes that affect the production of ACT, which is synthesized from acetyl coenzyme A (acetyl-CoA) and malonyl-CoA (Fig. 1A), a library with approximately 50,000 mutants was constructed in S. coelicolor M145 via pHL734-mediated transposon mutagenesis (36). pHL734 is a suicide plasmid; therefore, each mutant obtained by this method results from a single random transposition event (36). Under the conditions used, visual estimation of blue ACT production was not obscured by RED production, as ACT was excreted and RED production was relatively low. After phenotypic screening, we identified 410 mutants producing more blue pigment than the parental strain (M145), and 578 mutants producing less or no blue pigment, resulting in a total of 988 mutants for further study. The variations in ACT production of all mutants were quantified by measuring the absorbance at 633 nm (UV_633_) of alkaline extracts of cultures grown on yeast extract-beef extract-Bacto peptone (YBP) agar for 84 h. The mini-Tn*5* insertion sites of the selected 988 mutants were located by mini-Tn*5* rescuing and DNA sequencing. The insertion sites, inactivated genes or operons, and the relative ACT production levels of these 988 mutants are listed in Data Set S1 in the supplemental material.

Among the 988 mutants, 69 mutants had insertions within the 22.8-kb *act* gene cluster (Fig. 1B). Sixty-three of these mutants produced no or decreased ACT, and their insertion sites included the three minimal PKS genes *SCO5087* to *SCO5089* (*actI-orf1* to *actI-orf3*), the ketoreductase gene *SCO5086* (*actIII*), the aromatase gene *SCO5090* (*actVII*), the cyclase gene *SCO5091* (*actIV*), the transporter genes *SCO5076* (*actVA-orf1*) and *SCO5084* (*actII-orf3*), and the pathway-specific activator gene *SCO5085* (*actII-orf4*). One insertion in the TetR-like regulatory gene *SCO5082* (*actII-orf1*) increased ACT production as did an insertion in the ACT transporter gene *SCO5083* (*actII-orf2*). No insertion was found in *SCO5077* (*actVA2*) or *SCO5078* (*actVA3*), which have no assigned role in the ACT biosynthetic pathway. Eleven insertions were located within the 767-bp *actII-orf4* gene, a hit rate four times higher than the average hit rate for the *act* cluster genes. The high rate of insertion in *actII-orf4* might reflect the relatively low G+C content (63.5%) of this gene in comparison to that of the rest of the *act* cluster genes (36).

In addition to insertions in the *act* gene cluster, 919 insertions outside the *act* cluster were found to affect ACT production, including 478 insertions in 334 genes that decreased or abolished ACT production, 377 insertions in 229 genes that increased ACT production, and 27 and 37 mutants with increased or reduced ACT production, respectively, that had insertions in intergenic regions (Data Set S1). An analysis of the distribution of these 919 inserts within the chromosome indicated that there were more insertions in the chromosomal “core” region than in the “arm” regions (Fig. 1C and D).

### ACT regulatory genes outside the *act* gene cluster.

**FIG 2:**
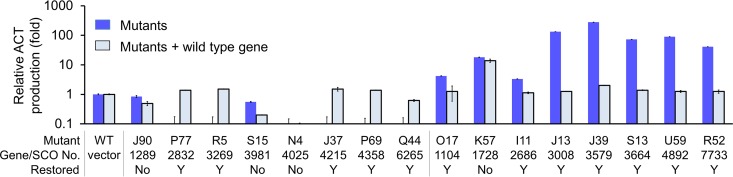
Relative ACT production in regulatory gene mutants and complemented strains. Antibiotic production levels in S. coelicolor M145 (WT) and M145/pMT3 (vector) are shown as a reference. Data are shown as the means from three experiments. Error bars indicate standard deviations. Y (yes) indicates that complementation restored parental ACT levels. No indicates that complementation did not restore parental ACT levels. Note that the decreased ACT production of mutants J90 and S15 was observable by eye.

Changes in ACT production by a mutant may be caused by spontaneous mutation. To evaluate the association between changes in ACT production and the insertions, 16 mutants with altered ACT production and which had inserts in genes of putative transcriptional regulators were selected from the library for in *trans* complementation. ACT production of 12 of these mutants was restored to the levels of strain M145 by the in *trans* complementation (Fig. 2), suggesting that changes in ACT production in about three-fourths of all of the mutants were attributable to the mini-Tn*5* insertions; the phenotypes of the others may be caused by unknown spontaneous mutation.

Furthermore, if several mutants had insertions in the same gene and similar changes in ACT production, then the findings would strongly indicate that the targeted gene had a role in ACT production. Peaks in the mutant distribution plot indicated multiple cases of insertions into the same target genes. In the distribution plot for mutants with decreased or abolished ACT production (Fig. 1C), the three highest peaks contained a cluster of vitamin B_12_ biosynthesis genes, the RNase J gene *SCO5745*, and the two consecutive genes *SCO2836* and *SCO2837*, which encode a putative glycosyl transferase and a galactose oxidase homolog, respectively, and which are reportedly involved in aerial mycelium development (37, 38). In addition, six genes, *SCO2241*, *SCO3025* (*manA*), *SCO4069* (*sarA*), *SCO4118* (*atrA*), *SCO4330*, and *SCO5204*, had more than five insertions, and among these genes, *manA*, *sarA*, and *atrA* have been reported to positively regulate ACT production (24, 39, 40). A total of 71 genes had more than one insertion associated with decreased ACT production implying that they are ACT upmodulators; among them, 55 have not been reported previously (see Table S1). A further 263 genes potentially associated with upmodulation of ACT were identified by single insertions.

The distribution plot of the 404 insertions associated with increased ACT production showed several high peaks (Fig. 1D). These peaks included the previously reported pleiotropic antibiotic negative-regulator gene *wblA* (*SCO3579*) (41), the putative ATP/GTP-binding membrane protein gene *SCO5677* (42), a cluster of membrane protein genes, *cmdBCDEF* (25), the putative secreted lytic transglycosylase gene *tgdA* (43), and the cytochrome *bd* oxidase and transporter genes *cydABCD*. In total, 50 genes had more than one insertion associated with increased ACT production, and 34 of these genes were not previously known to influence ACT production (Table S1). A further 179 potential downmodulators of ACT production were identified by single insertions (Data Set S1). Notably, there were 12 regulators, 5 transporters, and 14 “cell wall/membrane/envelope biogenesis” genes among the 71 ACT upmodulators for which there was more than one mutant. In addition, signaling/regulatory genes constituted 11 of the 50 downmodulators represented by more than one mutant (Table S1). Branched-chain amino acid biosynthesis genes, protein modification genes, DNA transfer genes, and cytochrome *bd* oxidase genes *cydABCD* were all found to modulate ACT production.

**FIG 3:**
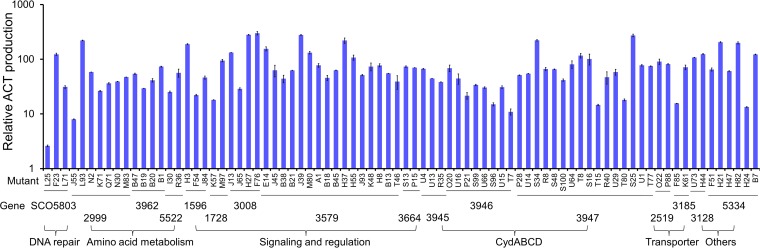
Mutants with increased ACT production. ACT production levels are shown relative to those in S. coelicolor M145. Bars indicate mutants with insertions in the same gene, and gene names and functional categories of the mutated genes are indicated below the mutants.

Remarkably, insertional mutations in some of the above-mentioned downmodulators increased ACT production >50-fold, with some mutants exhibiting increases of >200-fold. The insertion targets in these mutants included the following: five signaling and regulatory genes, *SCO1596* (*ohkA*), *SCO1728* (GntR family transcriptional regulator), *SCO3008* (two-component system response regulator), *SCO3579* (*wblA*), and *SCO3664* (regulator); the three amino acid metabolism genes *SCO3962* (*pheA*, encoding prephenate dehydratase), *SCO5522* (*leuB*, encoding 3-isopropylmalate dehydrogenase), and *SC2999* (encoding NAD-specific glutamate dehydrogenase); the two transporter genes *SCO2519* and *SCO3185*, encoding a putative antibiotic efflux protein and a potassium/proton antiporter, respectively; the DNA repair gene *SCO5803*; a putative fatty acid desaturase gene, *SCO3128*; and *SCO5334*, a gene of unknown function (Fig. 3).

**FIG 4:**
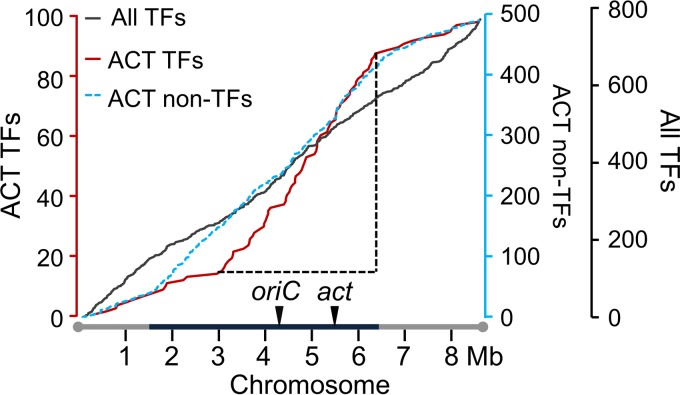
Distribution of transcriptional regulatory genes affecting the production of ACT along the S. coelicolor chromosome. Cumulative numbers are plotted. ACT TFs, transcriptional regulatory genes affecting ACT production. All regulatory genes present on the chromosome (All TFs) and nonregulator modulatory genes affecting ACT production (ACT non-TFs) are also plotted for reference. Regions showing a high density of ACT-modulating genes (increase in slope of graph) are indicated by dotted black lines. The chromosome replication initiation site (*oriC*) and the *act* genes are indicated by inverted triangles. TFs, transcriptional regulatory genes.

Transcriptional regulators were overrepresented among the mutated genes that affected ACT production. Of the 551 identified ACT modulators (Data Set S1), 110 were transcriptional regulators, including DNA binding proteins and sigma factors. Interestingly, the chromosomal distribution of the 110 ACT-modulatory transcriptional regulators showed a high density within a 3- to 6-Mb section of the core region of the linear chromosome, overlapping the ACT biosynthetic gene cluster and *oriC* (Fig. 4).

## DISCUSSION

In *Streptomyces*, antibiotic production is regulated by transcriptional regulators, substrate supply, cofactors, energy metabolism, reducing power, cell wall integrity, protein modification, etc. ACT biosynthesis is a model system for secondary metabolism research, and many genes have been implicated in the regulation of ACT production in S. coelicolor (33–35). To systematically survey genes affecting ACT production, we constructed a mutant library of S. coelicolor M145 using an efficient mini-Tn*5* transposition system, from which we identified 988 mutants with altered ACT production. A total of 570 genes were identified as possible modulators for ACT production, including 19 *act* genes.

Outside the ACT biosynthetic gene cluster, 121 genes were each disrupted in more than one mutant, and although the insertion positions differed for a given gene, the resulting mutants displayed similar phenotypes with regard to ACT production (see Table S1 in the supplemental material), strongly supporting these genes as modulators of ACT biosynthesis. Furthermore, in tests to confirm the association between changes in ACT and the sites of mini-Tn*5* insertion, the ACT production of 12 of 16 mutants was restored to almost parental levels by supplying wild-type genes. Based on these findings, we predict that approximately three-fourths of our detected mutants had altered ACT production due to the mini-Tn*5* insertions, whereas the remaining one-fourth of these mutants were false positives, potentially the result of spontaneous mutations that also affected ACT levels. If this prediction is correct, then among the 430 potentially ACT-modulating genes identified by single insertions, we anticipate that more than 300 of these genes can actually modulate ACT production.

**FIG 5:**
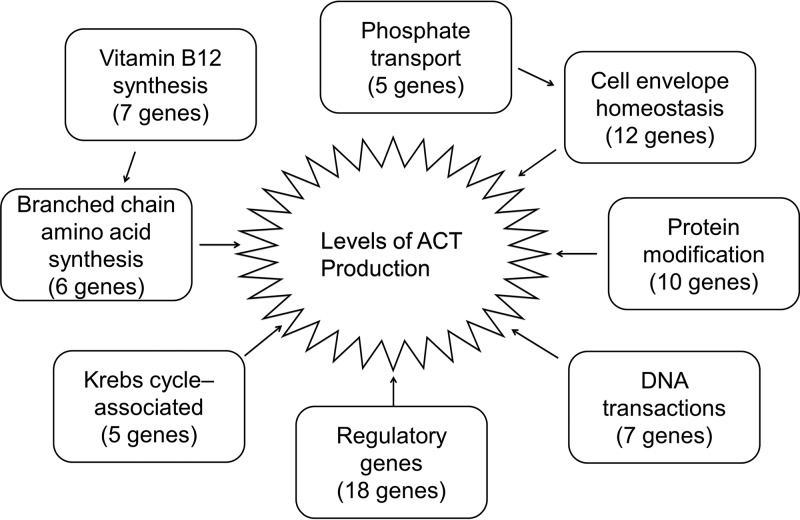
Overview of major classes of genes influencing ACT production. The following genes comprise the indicated sets: seven vitamin B_12_ biosynthesis genes, *SCO1848* to *SCO1850*, *SCO1852*, *SCO1853*, *SCO1855*, and *SCO1857*, are ACT upmodulators; six genes involved in branched-chain amino acid metabolism, including three ACT upmodulators, *SCO2528* (*leuA*), *SCO3345* (*ilvD*), and *SCO5512* (*ilvB*), and three ACT downmodulators, *SCO5513* (*ilvN*), *SCO5514* (*ilvC*), and *SCO5522* (*leuB*); five genes involved in the Krebs cycle, including one ACT upmodulator, *SCO5281*, and four ACT downmodulators, *SCO2999* and *SCO3945* to *SCO3947* (*cydABCD*); eighteen regulatory genes, including ten ACT upmodulators *SCO2792* (*adpA*), *SCO2832*, *SCO2987* (*ohrR*), *SCO3269*, *SCO3571*, *SCO3981*, *SCO4118*, *SCO4215*, *SCO4358*, and *SCO5351*, and eight ACT downmodulators, *SCO1728*, *SCO2179*, *SCO2686*, *SCO3008*, *SCO3579* (*wblA*), *SCO3664*, *SCO4426* (*afsR*), and *SCO5803* (*lexA*); seven DNA transaction genes, *SCO4127* to *SCO4131* (*cmdBCDEF*), *SCO5677*, and *SCO5803* (*lexA*), are ACT downmodulators; nine protein modification genes, including five ACT upmodulators, *SCO3025* (*manA*), *SCO3028* (*manB*), *SCO3154* (*pmt*), *SCO3404* (*ftsH2*), and *SCO4609*, and four ACT downmodulators, *SCO1388*, *SCO1646* (*pup*), *SCO1647*, and *SCO1648* (*arc*); twelve cell envelope homeostasis genes, including ten ACT upmodulators, *SCO1525*, *SCO2097*, *SCO2132*, *SCO2836* (*cslA*), *SCO2837* (*glxA*), *SCO3150* (*rpfB*), *SCO3899* (*inoA*), *SCO4440*, *SCO4878*, and *SCO5174*, and two ACT downmodulators, *SCO2085* (*ftsW*) and *SCO4132* (*tgdA*); and five phosphate transport-related genes, including three ACT upmodulators, *SCO3025* (*manA*), *SCO3028* (*manB*), and *SCO3154* (*pmt*), and two ACT downmodulators, *SCO1388* and *SCO4142* (*pstS*).

Our study revealed 570 genes that appeared to affect ACT biosynthesis under the conditions employed. Among them, 450 had not previously been reported as ACT modulators, and 176 of the genes encode hypothetical proteins or proteins of unknown function; approximately one-quarter (46/176) of these hypothetical protein-encoding genes were disrupted in more than one mutant. These findings presented a more integrative/global view of the regulation of ACT biosynthesis than was previously known. A comparison of the ACT modulators that had additional supporting evidence, i.e., more than one mutant or complementation with the wild-type gene, revealed several patterns (Table S1) (Fig. 5).

Branched-chain amino acid catabolism contributes to the production of large numbers of cellular metabolites (44). In our library, mutants of *SCO5513* (*ilvN*), *SCO5514* (*ilvC*), and *SCO5522* (*leuB*) had increased ACT production, whereas mutants of *SCO5512* (*ilvB*), *SCO3345* (*ilvD*), and *SCO2528* (*leuA*) had decreased ACT production. The *ilvB*, *ilvC*, *ilvD*, and *leuA* mutants were bald (deficient in developing aerial hyphae). Branched-chain amino acids are essential for protein biosynthesis and are also key intermediates in fatty acid biosynthesis in *Streptomyces*. In *Streptomyces*, more than 70% of fatty acids are branched chain with an ω-2 methyl group (45), and the biosynthesis of these fatty acids is initiated from the dehydrogenation of branched-chain amino acids that are converted to branched-chain α-keto acid starter units (46). Therefore, a reduction in the intracellular supply of branched-chain amino acids will decrease branched-chain fatty acid biosynthesis, freeing acetate-derived building blocks for ACT biosynthesis.

Mini-Tn*5* mutants of seven vitamin B_12_ biosynthesis genes had decreased ACT production, which may also be related to the supply of acetate-derived building blocks. Vitamin B_12_ is a cofactor required for the conversion of methylmalonyl-CoA to succinyl-CoA, which is the last step of branched-chain amino acid degradation and has been reported to provide 50% of the precursors for ACT biosynthesis (47). In addition, an accumulation of methylmalonyl-CoA in vitamin B_12_-deficient strains accelerates branched-chain fatty acid biosynthesis, again sparing acetyl-CoA precursors for ACT biosynthesis. However, vitamin B_12_ is a cofactor of other important enzymes involved in methionine biosynthesis, regeneration of the active cofactor tetrahydrofolate, and the formation of deoxyribonucleotides from ribonucleotides, and so other routes may also possibly contribute to the phenotypes of these mutants.

Mutants of *SCO2999*, which encodes an NAD^+^-specific glutamate dehydrogenase (GDH), had dramatically increased production of ACT. GDH converts glutamate to 2-oxo-glutarate, a key intermediate of the Krebs cycle, and boosts overall consumption of acetyl-CoA. Mutants of *SCO2999* might therefore be expected to weaken the Krebs cycle, making acetyl-CoA more readily available for ACT biosynthesis. *SCO2999* has been shown to affect growth and secondary metabolism in S. coelicolor (48). In our study, mutants of *SCO5281* had dark blue colonies, an indication of ACT overproduction; however, these mutants grew very poorly, so that the measured ACT yield per culture volume was reduced. *SCO5281* encodes an α-ketoglutarate decarboxylase, which is the E1 component of the α-ketoglutarate dehydrogenase complex in the Krebs cycle.

Mutants of the cytochrome *bd* oxidase genes *cydA* (*SCO3945*), *cydB* (*SCO3946*), and *cydCD* (*SCO3947*) also showed dramatically increased ACT production. In bacteria grown under hypoxic conditions, cytochrome *bd* terminal oxidase, which has high affinity for O_2_, takes the place of cytochrome *c* oxidase to restore and maintain efficient oxidative phosphorylation (49, 50), indirectly promoting the overall consumption of acetyl-CoA through the Krebs cycle. Thus, these mutants would have reduced consumption of acetyl-CoA, making more available for secondary metabolism.

Mutants of *tgdA* showed increased ACT production, to the same levels as the *cmdBCDEF* and *SCO5677* mutants, and these results were similar to those of our previous study showing that mutants of *tgdA*, *cmdBCDEF*, and *SCO5677* had increased RED production (36). TgdA, CmdBCDEF, and SCO5677 take part in the formation of aerial hyphae, implying that cell envelope biosynthesis/homeostasis affects antibiotic production.

Mutants of *inoA*, encoding *myo*-inositol-1-phosphate synthase, *SCO1525* and *SCO2132*, both of which encode phosphatidylinositol mannosyltransferase homologs, and *SCO4440*, encoding a homolog of eukaryotic phosphoinositide-binding signaling protein, showed decreased ACT production. In addition, mutants of *SCO2836* (*cslA*), *SCO4878*, *SCO5174*, and *SCO3150*, all encoding putative glycosyl-transferases and all of which are possibly involved in cell wall synthesis, also showed decreased ACT production. It has been reported that some ACT biosynthetic enzymes, i.e., ketoreductase and polyketide synthase α subunit, may be located in or outside the cell envelope (51, 52), suggesting that interference with membrane-related functions might affect the localization and maintenance of the active conformation of biosynthetic enzymes for compounds such as ACT. Alternatively, osmotic stress signal transduction, which requires an intact and reinforced cell envelope, might be disturbed in these mutants. This possibility is suggested by the finding that mutants of *SCO2837* (*glxA*), which encodes a galactose oxidase homolog required for aerial hyphal development during osmotic stress (38), and *SCO5748* (*osaA*), which encodes a sensory histidine kinase involved in osmotic adaptation (53), showed reduced ACT production.

Insertions in either *SCO3404* (*ftsH2*), encoding an ATP-dependent metalloprotease, or *SCO4609* (*htpX*), encoding a zinc metalloprotease HtpX homolog, reduced ACT production dramatically. FtsH and HtpX are involved in the proteolytic quality control of membrane protein processing, lending further support to the hypothesis that an intact cell envelope is important in ACT production. Insertions in genes involved in mannosylation, including *SCO1388*, encoding mannose-1-phosphate guanyltransferase, *SCO3025* (*manA*), *SCO3028* (*manB*), and SCO3154 (*pmt*) all showed decreased ACT production. Mannosylation of the high-affinity phosphate-binding protein PstS by Pmt has been reported in *Streptomyces* (54), and insertions in *SCO4142* (*pstS*) increased ACT production, suggesting that the decreased ACT production seen in the mannosylation mutants may be, at least in part, due to altered regulation of phosphate uptake. In addition, as a *pmt* mutant lost intrinsic functions of the cell envelope (55), the decreased ACT production of our *pmt* mutant may be caused by cell envelope damage.

Mutants in the pupylation genes *SCO1646*, encoding Pup protein, a prokaryotic ubiquitin-like protein, *SCO1647* (a Pup ligase), and *SCO1648* (an AAA ATPase) increased ACT production, in agreement with a previous report (56). However, this finding may reflect involvement of the Pup system in oxidative stress rather than its better-understood role in protein degradation, since a mutation in the proteasome gene showed only modest perturbations in ACT production (56) and no proteasome mutants were identified in our experiments.

Many previously reported genes involved in stress response, signal transduction, and transcriptional regulation were found in our study to modulate ACT production, including genes involved in cAMP signaling (*SCO4928* and *SCO3571*) (57, 58), oxidative stress (*SCO2987*) (59), osmotic stress (*SCO5748*) (53), and transcriptional regulation (*wblA*, *sarA*, *atrA*, *afsR*, *rsfA*, *SCO3269*, and *SCO3723*) (21, 24, 41, 43, 60–63). Some of these proteins regulate antibiotic production by changing the expression of the biosynthetic gene clusters, e.g., *atrA*, *nsdA*, *SCO2179*, and *wblA* (41, 43, 64, 65), or activation of the substrate, e.g., *SCO1596* (66).

Other ACT-modulating genes did not fall into the above categories, including some that showed dramatically increased antibiotic production when inactivated. For example, mutants of *SCO5334*, encoding a hypothetical protein, and *SCO3128*, encoding a putative fatty acid desaturase, increased ACT production >100-fold. These genes are particularly important new targets for future studies on antibiotic production mechanisms and strain engineering.

## MATERIALS AND METHODS

### Plasmid, strains, primers, and culture conditions.

Vector pHL734 (GenBank accession number KU672723) was used for transposition mutagenesis in *Streptomyces* (36), and pMT3 was used as the delivery vector for genetic complementation (36). Escherichia coli ET12567/pUZ8002 was used as the donor for E. coli/S. coelicolor conjugation (67). The S. coelicolor M145 strain was used to construct the mutagenesis library. All primers used in this study are listed in Table 1. Culture conditions of strains have been described previously (36). Briefly, E. coli strains were grown in Luria-Bertani (LB) broth, *Streptomyces* strains were grown on mannitol soya flour (MS) agar medium for sporulation and conjugation, on YBP agar medium for ACT production screening, and in yeast extract-malt extract (YEME) medium for genomic DNA extraction.

**TABLE 1:** Primers used in this study

Primer	Primer sequence (5′→3′)
DownS	ACTGCTGTGAGCGCTTTGCCTTGGC
UpS	ATAAACTTATCATCCCCTTTTGCTG
SCO1728-F	TCGGGATGGACGTAGACGTTCAG
SCO1728-R	CCGCGGCCCCGATCCCGACGGCG
SCO2686-F	CCGTCGAATCGGTGAAGGCAGAG
SCO2686-R	GACTACACCTCGCTCCGTCGGCG
SCO2832-F	TCCTGGACCTGCTGCGGGACATC
SCO2832-R	CAGTGCCCGGGCGGTCCTAGATC
SCO3981-F	CGCCCAAAGGGTGCGGCCGTTCA
SCO3981-R	CCTTGGACTCGGCGTAGAGAATC
SCO4358-F	GCCCGCCACCTCGGCCCGTCCCC
SCO4358-R	CGTCGCGCTGGCCGCGCTGTTCG
SCO4892-F	CGCCGTAGCAGGTCTGCGCCGTG
SCO4892-R	GGCGCCGGCCCGGTAGGGTCCCG

### Mutagenesis of S. coelicolor strains using pHL734.

As reported previously (36), transposon vector pHL734 was introduced into S. coelicolor M145 by conjugation from E. coli ET12567 containing pUZ8002. After trimethoprim (50 μg · ml^−1^) and apramycin (50 μg · ml^−1^) flooding, transposon mutants were obtained from the conjugation plates and incubated for 5 days at 30°C, and then these exconjugants were transferred to solid YBP medium to screen for ACT production.

### Identification of insertion sites in transposon mutants.

Identification of insertion sites was conducted as previously described (36). Briefly, the genomic DNA of transposon mutants was extracted and digested with ApaI, and the digested product was self-ligated and introduced into E. coli DH5α. Apramycin-resistant colonies were selected, and the recombinant plasmid was sequenced with primers DownS and/or UpS.

### Measurement of ACT production.

*Streptomyces* strains were cultured on YBP medium for 84 h at 30°C, and 500 mg agar medium (containing both bacterium and agar) of each culture was excised from the plates. The collected agar culture was dispersed into 500 μl of 1 M NaOH using a homogenizer (twice at 5,000 rpm for 15 s). The samples were centrifuged at 12,000 × *g* for 5 min to remove the particulate matter, and the absorbance of the supernatants at 633 nm was measured (68). The relative ACT production of each mutant was calculated from the ratio *A*_633_ mutant/*A*_633_ wild type. Data are representative of two independent experiments.

### Genetic complementation.

For genetic complementation of the mini-Tn*5* insertion mutants, the promoter and coding region of the target gene were amplified from the S. coelicolor M145 chromosome using appropriate primers (Table 1), confirmed by DNA sequencing, and then cloned into the integrative vector pMT3. The *cis* complementation plasmids (Table 2) were transformed into the appropriate S. coelicolor mutants by conjugation, and clones containing complementing copies of the wild-type genes were obtained by selection on thiostrepton (20 μg · ml^−1^).

**TABLE 2:** Plasmids used in this study

Plasmid	Description	Reference or source
pHL734	Mini-Tn*5* transposon vector, *amp*^r^, *apr*^r^	36
pMT3	*Streptomyces* integrative vector, *thio*^r^	36
pHXZ097	pMT3 harboring *SCO1104*, for gene complementation	36
pHXZ098	pMT3 harboring *SCO1289*, for gene complementation	36
pHXZ099	pMT3 harboring *SCO1728*, for gene complementation	This work
pHXZ101	pMT3 harboring *SCO2686*, for gene complementation	This work
pHXZ102	pMT3 harboring S*CO2832*, for gene complementation	This work
pHXZ103	pMT3 harboring S*CO3008*, for gene complementation	36
pHXZ104	pMT3 harboring S*CO3269*, for gene complementation	36
pHXZ105	pMT3 harboring S*CO3579*, for gene complementation	36
pHXZ106	pMT3 harboring S*CO3664*, for gene complementation	36
pHXZ107	pMT3 harboring *SCO3981*, for gene complementation	36
pHXZ108	pMT3 harboring *SCO4025*, for gene complementation	36
pHXZ109	pMT3 harboring *SCO4215*, for gene complementation	36
pHXZ110	pMT3 harboring *SCO4358*, for gene complementation	This work
pHXZ111	pMT3 harboring *SCO4892*, for gene complementation	This work
pHXZ114	pMT3 harboring *SCO6265*, for gene complementation	36
pHXZ115	pMT3 harboring *SCO7733*, for gene complementation	36

## Supplementary Material

Supplemental file 1

Supplemental file 2
