# Supplementary material for: Genome-Wide Mutagenesis Links Multiple Metabolic Pathways with Actinorhodin Production in Streptomyces coelicolor
Source: Appl Environ Microbiol. 2019 Mar 22;85(7):e03005-18. doi: 10.1128/AEM.03005-18 (PMC6585502; doi:10.1128/AEM.03005-18)
Supplement: Supplemental file 1 [file AEM.03005-18-s0001.pdf]

TABLE S1 Genes with multiple transposon inserted mutants affecting ACT production (= reliable ACT modulatory genes)

Function group and Protein ID	Length (aa)	Annotation and description [a]	No. of Mutants	ACT production by mutants compared to parent strain [b]	Report ACT-modulation
**Morphological development [c]**					
SCO2792	398	AdpA/BldH, AraC family transcription regulator. AdpA binds *redD* promoter (1) and also represses the expression of the antibiotic negative regulator *wblA* (2).	2	0.7, 0.3	(2)
**Cell envelope biosynthesis**					
SCO1525	387	Probable phosphatidylinositol mannosyltransferase, 54% identity with PimA which catalyses the transfer of mannose to phosphatidylinositol to form phosphatidylinositol mono-mannoside in mycobacteria (3).	2	0.1, 0.1	This study
SCO2097	135	Conserved actinomycetes membrane protein associated with division cell wall synthesis (4).	2	0.6, 0.4	This study
SCO2132	412	Possible phosphatidylinositol mannosyltransferase.	3	0.4, 0, 0.4	This study
SCO2836	653	Glycosyl transferase CslA, located in hyphal tips, associated with a β(1-4) glucan biosynthesis, required for aerial hyphae development (5).	5	0, 0.9, 0.7, 0.4, 0.6	(5)
SCO2837	645	GlxA, secreted metal dependent galactose oxidase, required for aerial development during osmotic stress (6, 7).	4	0, 0, 0.3, 0	(6, 7)
SCO3150	458	RpfB, a cell wall-lytic enzyme. *rpfB* mutant is sensitive to cell wall specific antibiotics (8).	2	0.6, 0.7	This study
SCO3899	360	InoA, myco-inositol-1-phosphate synthase (mIPS) (9).	3	0.6, 0.6, 0.7	(9)

Table S1 continued

SCO4440	252	Homologue of the conserved oncoprotein Golgi phosphoprotein 3 involved in phosphoinositide signaling and regulation of multiple cellular functions (10).	3	0.3, 0.6, 0.3	This study
SCO4878	326	Putative glycosyltransferase in cell wall biosynthesis.	2	0.9, 0.9	This study
SCO5174	593	Putative glycosyltransferase in cell wall biosynthesis.	2	0.7, 0.6	This study
SCO2085	456	FtsW, cell division protein required for stabilize the Z ring during sporulation (11, 12).	3	2.5, 5.2, 6.2	(11, 12)
SCO4132	340	TgdA, putative secreted lytic transglycosylase. Mutant of *tgdA* increased ACT production that coincided with the overexpression of *actII*-*orf4* (13).	5	11.9, 18.2, 19.2, 17.5, 19.1	(13)
**DNA replication, repair, and transfer**					
SCO4127	471	CmdB, putative type IV secretion system protein TraC/VirB4. *cmdB* belongs to the *cmdA*-*F* membrane protein gene cluster. Mutant of *cmdA*-*F* displayed aberrant hyphae branches, abnormal chromosome segregation and spore septation, increased ACT production, and increased *actII*-*orf4* transcription (14).	5	29.4, 9.6, 7.3, 11.2, 25.9	(14)
SCO4128	513	CmdC, hypothetical protein. *cmdC* belongs to the *cmdA*-*F* membrane protein gene cluster (14).	11	27.2, 17.3, 33.0, 21.1, 8.4, 26.6, 9.2, 20.5, 7.4, 33.8, 8.3	(14)
SCO4129	420	CmdD, possible integral membrane protein. *cmdD* belongs to the *cmdA*-*F* membrane protein gene cluster (14).	2	27.4, 11.2	(14)
SCO4130	266	CmdE, hypothetical protein. *cmdE* belongs to the *cmdA*-*F* membrane protein gene cluster (14).	2	17.9, 17.5	(14)
SCO4131	102	CmdF, membrane protein. *cmdF* belongs to the *cmdA*-*F* membrane protein gene cluster (14).	3	12.9, 19.1, 37.8	(14)

Table S1 continued

SCO5677	676	Putative type IV secretion system protein TraC/VirB4.	17	4.8, 0.6, 13.8, 8.0, 8.0, 24.2, 17.5, 4.2, 3.9, 30.6, 32.8, 15.7, 7.1, 3.0, 8.6, 10.0, 4.1	(15)
SCO5803	234	LexA, SOS regulatory protein, repress a number of genes involved in the response to DNA damage (16).	4	2.6, 123.2, 31.1, 8.0	This study
**RNA processing**					
SCO4039	182	Putative tRNA adenosine deaminase-associated protein.	3	0.4, 0.3, 0.3	This study
SCO5745	561	RNase J, possessing both endo- and exonucleolytic activities. Mutants of SCO5745 delayed ACT production and increased RED production (17).	12	0.9, 0.1, 0.3, 0.3, 0.4, 0.4, 0.7, 0.3, 0.4, 0.4, 0.1, 0.3	(17)
SCO2793	200	OrnA, oligoribonuclease, hydrolyzes ribonucleic acid oligomers fewer than eight nucleotides. *ornA* mutant was unable to form aerial hyphae and increased ACT production in *S. coelicolor* (18).	5	13.1, 5.4, 12.4, 11.5, 11.8	(18)
**Protein modification**					
SCO3025	383	ManA, mannose-6-phosphate isomerase, interconverts fructose-6P and mannose-6P, mutant of *manA* failed to produce ACT and RED in liquid media (19).	6	0.7, 0.4, 0, 0.3, 0.1, 0	(19)
SCO3028	454	ManB, phosphomannomutase/phosphoglucomutase, catalyzes the reversible conversion of mannose-6P and mannose-1P. Mutant of *manB* increased ACT production in low-glucose media (20).	2	0.1, 0.3	(20)
SCO3154	591	Putative protein O-mannosyltransferase Pmt, glycosylation the PstS proteins in *S. coleicolor* (21).	4	0.4, 0.3, 0.4, 0.1	(21)
SCO3404	668	FtsH2, ATP-dependent metalloprotease required for cell division in bacteria.	2	0, 0.7	This study

Table S1 continued

SCO4609	287	Similar to protease HtpX in *E. coli*, which is a putative membrane-bound zinc metalloprotease, involved in the proteolytic quality control of membrane proteins (22).	3	0, 0, 0	This study
SCO1388	831	Mannose-1-phosphate guanyltransferase, involved in early steps of protein glycosylation.	2	10.5, 11.3	This study
SCO1646	71	Pup, prokaryotic ubiquitin-like protein, first detected in *M. tuberculosis*. Pup protein covalently conjugated to the ε-NH2 groups of lysines on the target proteins marking them for degradation (23, 24).	3	4.1, 3.2, 6.7	This study
SCO1647	497	Pup-ligase protein.	4	3.2, 12.5, 6.0, 0.3	This study
SCO1648	588	Arc, AAA ATPase forming ring-shaped complexes associated with 20S proteasome.	2	3.6, 2.6	This study
**Amino acid metabolism**					
SCO2241	453	GlnA2, glutamine synthetase homologue lacking highly conserved residues involved in catalysis (25).	5	0.1, 0.6, 0.1, 0, 0	This study
SCO2528	574	LeuA, 2-isoprylmalate synthase.	3	0, 0.45, 0.41	This study
SCO3345	617	IlvD, dihydroxy-acid dehydratase involved in the biosynthesis of valine and isoleucine.	2	0, 0.1	This study
SCO5281	1272	α-ketoglutarate decarboxylase.	3	0.4, 0.6	This study
SCO5512	614	acetolactate synthase 1 catalytic subunit	4	0, 0.27, 0.42, 0.85	This study
SCO2999	1653	NAD-specific glutamate dehydrogenase.	6	220.5, 58.1, 26.3, 36.1, 39.0, 47.2	This study
SCO3962	309	PheA, prephenate dehydratase.	4	54.0, 29.3, 41.2, 73.2	This study
SCO5513	174	IlvN, acetolactate synthase 3 regulatory subunit.	2	6.5, 4.6	This study
SCO5514	332	IlvC, acetolactate synthase small subunit.	2	7.4, 10.2	This study

Table S1 continued

SCO5522	347	LeuB, 3-isopropylmalate dehydrogenase.	2	25.2, 56.3	This study
**Stress, signal, and transcription regulator**					
SCO0871	531	Two-component sensor protein.	2	0.9, 0.6	This study
SCO1663	409	MshC, cysteinyl-tRNA synthetase involved in mycothiol biosynthesis (26).	2	0.6, 0	This study
SCO1867	280	EctD, ectoine hydroxylase catalyzes the biosynthesis of 5-hydroxyectoine from ectoine. Both ectoine and hydroxyectoine are compatible solutes against salt stress (27).	2	0.1, 0	(27)
SCO2832	285	IclR family transcriptional regulator.	2	0.1, 0	This study
SCO2987	168	OhrR, MarR family regulators response to organic peroxide stress (28).	4	0, 0.9, 0, 0.3	This study
SCO3269	251	GntR family regulator, regulates ACT production and morphological differentiation.	4	0.6, 0, 41.2, 0.1	(29)
SCO3571	224	CRP, cAMP binding domain containing regulator (30).	2	0.4, 0.4	This study
SCO3723	498	Negative regulator for ACT production (31).	2	0.3, 0.4	(31)
SCO3981	251	GntR family transcriptional regulator.	2	0.6, 0.3	This study
SCO4069	664	SarA, sporulation and antibiotic production related gene A, membrane protein. *sarA* mutant accelerated sporulation and dramatically decreased the production of ACT and RED (32).	7	0.6, 0, 0, 0.6, 0, 0, 0.3	(32)
SCO4118	297	AtrA, TetR family transcriptional regulator, positively regulates ACT production by directly binding to the promoter of *actII-orf4* (33).	7	0.3, 0, 0.3, 0, 0, 0.3, 0	(33)
SCO4204	496	MshA, glycosyltransferase invovled in mycothiol biosynthesis (26).	5	0, 0.6, 0, 0.1	This study
SCO4215	252	GntR family regulatory protein.	3	0.7, 0.6, 0	This study

Table S1 continued

SCO4358	268	TetR family transcriptional regulatory protein.	2	0, 0	This study
SCO4928	381	Adenylate cyclase Cya, synthesizes cAMP.	3	0, 0.9, 0.3, 0.7	(34)
SCO5351	167	Deletion mutant of SCO5351 reduced ACT production (35).	2	0.1, 0.1	(35)
SCO5748	1829	OsaA, sensory histidine kinase involved in osmotic adaptation.	2	0.4, 0.6	(36)
SCO6994	1086	TPR domain containing protein.	4	0, 0, 0, 0	This study
SCO1596	379	OhkA, orphan histidine kinase, disrupted mutant increases productions of ACT and RED owing to increased acetyl-CoA carboxylase transcription (37).	3	188.3, 22.1, 46.3	(37)
SCO1728	134	GntR family transcriptional regulator.	2	18.0, 94.2	This study
SCO2179	517	Leucyl aminopeptidase homologue without aminopeptidase activity, negatively affects the ACT production and sporulation, mutant increases transcription of *ftsZ, ssgA, metK*, and *actII-orf4* (38).	5	7.4, 7.8, 7.8, 8.6, 21	(38)
SCO2686	338	LuxR family transcriptional regulator.	2	3.3, 15.7	This study
SCO3008	252	Two-component system response regulator.	4	132.1, 28.7, 279.9, 299.5	This study
SCO3579	112	WblA, Wbl family regulator, pleiotropic down-regulator for antibiotic production in many streptomycetes (39-42).	17	154.1, 62.6, 155.1, 44.0, 62.5, 131.5, 278.3, 73.2, 51.2, 107.0, 219.6, 62.7, 45.6, 77.3, 77.2, 55.0, 39.0	(39)
SCO3664	336	Regulatory protein.	2	73.2, 70	This study
SCO4426	993	AfsR, global positive regulator of secondary metabolism in *Streptomyces*. AfsR actives the expression of sigma-like protein encoding gene *afsS* and competes with phosphate regulator PhoP for the expression *afsS*, *pstS,* and *phoRP* (43, 44).	2	35.7, 11.1	(43, 44)

Table S1 continued

SCO4677	114	RsfA, SigF-specific anti-sigma factor, anti-anti-sigma factor BldG releases the SigF from RsfA, the anti-sigma factor binding abilities of BldG could be inactivated by phosphorylation by RsfA (45). Mutant of *rsfA* increased ACT production (46).	2	10.2, 27.9	(46)
SCO5220	326	TPR domain containing protein, may be involved in signal transduction.	2	27.9, 14.7	This study
SCO5582	500	NsdA, negatively regulates *actII-orf4* expression (47).	3	6.1, 2.5, 8.1	(47)
SCO6636	974	PglZ, bacteriophage resistance protein (48).	2	7.1, 7.4	This study
**Respiratory chain**					
SCO3945	501	CydA, cytochrome oxidase subunit I,	3	66.8, 44.3, 38.3	This study
SCO3946	334	CydB, cytochrome oxidase subunit II.	8	68.6, 44.3, 21.5, 33.8, 30.3, 15.0, 30.9, 10.9	This study
SCO3947	1172	CydCD, ABC transporter.	16	51.5, 54.4, 221.5, 66.1, 65.5, 41.5, 80.8, 116.3, 101.0, 14.5, 46.9, 58.2, 18.0, 272.3, 77.3, 74.8	This study
**Secondary metabolism**					
SCO0381	491	Putative deoxysugar/glycosyltransferase.	2	0.3, 0.4	This study
SCO0390	410	Hypothetical protein.	2	0.6, 0.3	This study
SCO0395	341	Putative epimerase/dehydratase.	3	0, 0.3, 0.6	This study
SCO5315	159	WhiE-ORFVI, polyketide cyclase, involved in gray spore pigment biosynthesis in *S. coelicolor* (49, 50).	2	0.6, 0.9	This study
SCO5319	157	WhiE-ORFII, involved in gray spore pigment biosynthesis in *S. coelicolor* (49).	2	0.1, 0.4	This study
SCO5882	395	RedV, in RED biosynthetic gene cluster.	2	13.1, 16.0	This study

Table S1 continued

SCO5884	296	RedT, in RED biosynthetic gene cluster.	3	10.5, 14.8, 0.3	This study
SCO5892	2297	RedL, polyketide synthase for RED biosynthesis.	5	18.3, 17.6, 43.7, 31.3, 39.7	This study
SCO3230	7463	CDA peptide synthetase I.	2	44.4, 12.1	This study
**Transporter**					
SCO2254	413	Transmembrane efflux protein.	2	0.7, 0.4	This study
SCO2534	434	Similar to Mg^{2+} and Co^{2+} transporter CorB.	3	0.1, 0.1, 0	This study
SCO3765	459	Similar to Mg^{2+} and Co^{2+} transporter CorB.	3	0.6, 0.6, 0.3	This study
SCO6160	795	Bifunctional preprotein translocase subunit SecD/SecF.	4	0.3, 0.1, 0, 0.4	This study
SCO2519	692	Homologue of the transmembrane transport protein MmpL in *M. tuberculosis*, involved in the lipid transport and antibiotic efflux (51).	2	90.3, 81.0	This study
SCO3185	515	Potassium/proton antiporter.	2	15.6, 71.3	This study
**Vitamin B12 biosynthesis**					
SCO1848	502	Cobyric acid synthase.	2	0.6, 0.3	This study
SCO1849	1217	Cobaltochelatase subunit CobN.	4	0, 0.7, 0.3, 0.7	This study
SCO1850	672	Chelatase.	2	0, 0.6	This study
SCO1852	486	Cobyrinic acid a, c-diamide synthase.	2	0.1, 0	This study
SCO1853	243	Precorrin-2 C20-methyltransferase.	2	0.3, 0.4	This study
SCO1855	281	Precorrin-4 C11-methyltransferase.	2	0.3, 0.4	This study
SCO1857	583	Bifunctional protein (CbiGH).	2	0, 0.1	This study
**Others**					
SCO0700	589	ABC transporter protein ATP-binding protein.	2	0.4, 0	This study
SCO3176	583	Hypothetical protein.	2	0.4, 0.7	This study
SCO3294	448	Transferase.	2	0.1, 0.4	This study
SCO3324	419	Hypothetical protein.	3	0.3, 0.9, 0.1	This study

Table S1 continued

SCO3349	271	Hypothetical protein.	2	0, 0.7	This study
SCO3963	445	Homologue of Dyp-type peroxidase.	2	0.4, 0.6	This study
SCO3968	284	Integral membrane protein.	3	0.4, 0.7, 0	This study
SCO4106	376	RibD, putative bifunctional enzyme deaminase/reductase, involved in riboflavin biosynthesis.	2	0.3, 0.4	This study
SCO4176	280	Hypothetical protein.	2	0.1, 0.1	This study
SCO4330	308	Hypothetical protein.	5	0.1, 0.4, 0.4, 0.6, 0.1	This study
SCO5204	1000	Integral membrane protein.	6	0.3, 0.6, 0, 0, 0, 0.7	This study
SCO5546	261	Hypothetical protein.	2	0.9, 0.6	This study
SCO5771	132	Contains a polysulfide sulfurtransferase-rhodanese homology domain involved in signaling (52).	2	0.7, 0.6	(15)
SCO5954	765	Chitinase (secreted protein).	2	0.4, 0	This study
SCO5979	275	Enoyl-CoA hydratase.	2	0.3, 0.9	This study
SCO1307	468	Hypothetical protein.	2	9.7, 7.7	This study
SCO1901	329	Zinc-binding dehydrogenase.	4	13.5, 15, 8.6, 23.3	This study
SCO2365	245	Uncharacterized protein involved in tellurium resistance.	2	20.2, 21.2	This study
SCO3096	426	Eno, enolase, catalyzes the reversible dehydration of 2-phospho-D-glycerate to phosphoenolpyruvate.	2	45.4, 32.0	This study
SCO3128	336	Fatty acid desaturase (membrane).	2	107.5, 123.9	This study
SCO3656	185	Hypothetical protein.	2	24.9, 14.7	This study
SCO3663	63	Hypothetical protein.	3	5.2, 2.2, 4.2	This study
SCO4142	370	Phosphate-binding protein precursor PstS, mutant of *pstS* increased ACT production on R2YE medium (53).	3	43.8, 8.0, 13.1	(53)
SCO4175	130	Hypothetical protein.	3	28.4, 5.4, 9.9	This study

Table S1 continued

SCO5334	104	Hypothetical protein.	6	122.0, 13.4, 64.8, 204.6, 60.9, 198.9	This study

a, annotation and description taken from StrepDB (http://strepdb.streptomyces.org.uk) or literatures. b, mean value of each mutant.
c, prviously identified morphological development genes. *S. coelicolor* does not from grey spores when grown on YBP media used in this study.

1. **Park SS, Yang YH, Song E, Kim EJ, Kim WS, Sohng JK, Lee HC, Liou KK, Kim BG.** 2009. Mass spectrometric screening of transcriptional regulators involved in antibiotic biosynthesis in *Streptomyces coelicolor* A3(2). J Ind Microbiol Biotechnol **36:**1073-1083. http://dx.doi.org/10.1007/s10295-009-0591-2.
2. **Lee HN, Kim JS, Kim P, Lee HS, Kim ES.** 2013. Repression of antibiotic downregulator WblA by AdpA in *Streptomyces coelicolor*. Appl Environ Microbiol **79:**4159-4163. http://dx.doi.org/10.1128/aem.00546-13.
3. **Kordulakova J, Gilleron M, Mikusova K, Puzo G, Brennan PJ, Gicquel B, Jackson M.** 2002. Definition of the first mannosylation step in phosphatidylinositol mannoside synthesis. PimA is essential for growth of *mycobacteria*. J Biol Chem **277:**31335-31344. http://dx.doi.org/10.1074/jbc.M204060200.
4. **Kleinschnitz EM, Heichlinger A, Schirner K, Winkler J, Latus A, Maldener I, Wohlleben W, Muth G.** 2011. Proteins encoded by the *mre* gene cluster in *Streptomyces coelicolor* A3(2) cooperate in spore wall synthesis. Mol Microbiol **79:**1367-1379. http://dx.doi.org/10.1111/j.1365-2958.2010.07529.x.
5. **Xu H, Chater KF, Deng Z, Tao M.** 2008. A cellulose synthase-like protein involved in hyphal tip growth and morphological differentiation in *streptomyces*. J Bacteriol **190:**4971-4978. http://dx.doi.org/10.1128/jb.01849-07.
6. **Whittaker MM, Whittaker JW.** 2006. *Streptomyces coelicolor* oxidase (SCO2837p): a new free radical metalloenzyme secreted by *Streptomyces coelicolor* A3(2). Arch Biochem Biophys **452:**108-118. http://dx.doi.org/10.1016/j.abb.2006.06.020.
7. **Liman R, Facey PD, van Keulen G, Dyson PJ, Del Sol R.** 2013. A laterally acquired galactose oxidase-like gene is required for aerial development during osmotic stress in *Streptomyces coelicolor*. PLoS One **8:**e54112. http://dx.doi.org/10.1371/journal.pone.0054112.
8. **Sexton DL, St-Onge RJ, Haiser HJ, Yousef MR, Brady L, Gao C, Leonard J, Elliot MA.** 2015. Resuscitation-promoting factors are cell wall-lytic

enzymes with important roles in the germination and growth of *Streptomyces coelicolor*. J Bacteriol **197:**848-860. http://dx.doi.org/10.1128/jb.02464-14.

9. **Zhang G, Tian Y, Hu K, Zhu Y, Chater KF, Feng C, Liu G, Tan H.** 2012. Importance and regulation of inositol biosynthesis during growth and differentiation of *Streptomyces*. Mol Microbiol **83:**1178-1194. http://dx.doi.org/10.1111/j.1365-2958.2012.08000.x.
10. **Sechi S, Frappaolo A, Belloni G, Colotti G, Giansanti MG.** 2015. The multiple cellular functions of the oncoprotein Golgi phosphoprotein 3. Oncotarget **6:**3493-3506. http://dx.doi.org/10.18632/oncotarget.3051.
11. **Mistry BV, Del Sol R, Wright C, Findlay K, Dyson P.** 2008. FtsW is a dispensable cell division protein required for Z-ring stabilization during sporulation septation in *Streptomyces coelicolor*. J Bacteriol **190:**5555-5566. http://dx.doi.org/10.1128/jb.00398-08.
12. **Bennett JA, Yarnall J, Cadwallader AB, Kuennen R, Bidey P, Stadelmaier B, McCormick JR.** 2009. Medium-dependent phenotypes of *Streptomyces coelicolor* with mutations in *ftsI* or *ftsW*. J Bacteriol **191:**661-664. http://dx.doi.org/10.1128/jb.01048-08.
13. **Xie P, Zeng A, Lv X, Cheng Q, Qin Z.** 2013. A putative transglycosylase encoded by *SCO4132* influences morphological differentiation and actinorhodin production in *Streptomyces coelicolor*. Acta Biochim Biophys Sin (Shanghai) **45:**296-302. http://dx.doi.org/10.1093/abbs/gmt012.
14. **Xie P, Zeng A, Qin Z.** 2009. *cmdABCDEF*, a cluster of genes encoding membrane proteins for differentiation and antibiotic production in *Streptomyces coelicolor* A3(2). BMC Microbiol **9:**157. http://dx.doi.org/10.1186/1471-2180-9-157.
15. **Gehring AM, Nodwell JR, Beverley SM, Losick R.** 2000. Genomewide insertional mutagenesis in *Streptomyces coelicolor* reveals additional genes involved in morphological differentiation. Proc Natl Acad Sci U S A **97:**9642-9647. http://dx.doi.org/10.1073/pnas.170059797.
16. **Harmon FG, Rehrauer WM, Kowalczykowski SC.** 1996. Interaction of *Escherichia coli* RecA protein with LexA repressor. II. Inhibition of DNA strand exchange by the uncleavable LexA S119A repressor argues that recombination and SOS induction are competitive processes. J Biol Chem **271:**23874-23883.
17. **Bralley P, Aseem M, Jones GH.** 2014. SCO5745, a bifunctional RNase J ortholog, affects antibiotic production in *Streptomyces coelicolor*. J Bacteriol **196:**1197-1205. http://dx.doi.org/10.1128/jb.01422-13.
18. **Sello JK, Buttner MJ.** 2008. The oligoribonuclease gene in *Streptomyces coelicolor* is not transcriptionally or translationally coupled to *adpA*, a key *bldA* target. FEMS Microbiol Lett **286:**60-65. http://dx.doi.org/10.1111/j.1574-6968.2008.01260.x.
19. **Rajesh T, Song E, Kim JN, Lee BR, Kim EJ, Park SH, Kim YG, Yoo D, Park HY, Choi YH, Kim BG, Yang YH.** 2012. Inactivation of phosphomannose isomerase gene abolishes sporulation and antibiotic production in *Streptomyces coelicolor*. Appl Microbiol Biotechnol **93:**1685-1693. http://dx.doi.org/10.1007/s00253-011-3581-z.

20. **Yang YH, Song E, Park SH, Kim JN, Lee K, Kim E, Kim YG, Kim BG.** 2010. Loss of phosphomannomutase activity enhances actinorhodin production in *Streptomyces coelicolor*. Appl Microbiol Biotechnol **86:**1485-1492. http://dx.doi.org/10.1007/s00253-009-2368-y.
21. **Wehmeier S, Varghese AS, Gurcha SS, Tissot B, Panico M, Hitchen P, Morris HR, Besra GS, Dell A, Smith MC.** 2009. Glycosylation of the phosphate binding protein, PstS, in *Streptomyces coelicolor* by a pathway that resembles protein O-mannosylation in eukaryotes. Mol Microbiol **71:**421-433. http://dx.doi.org/10.1111/j.1365-2958.2008.06536.x.
22. **Sakoh M, Ito K, Akiyama Y.** 2005. Proteolytic activity of HtpX, a membrane-bound and stress-controlled protease from *Escherichia coli*. J Biol Chem **280:**33305-33310. http://dx.doi.org/10.1074/jbc.M506180200.
23. **Pearce MJ, Mintseris J, Ferreyra J, Gygi SP, Darwin KH.** 2008. Ubiquitin-like protein involved in the proteasome pathway of *Mycobacterium tuberculosis*. Science **322:**1104-1107. http://dx.doi.org/10.1126/science.1163885.
24. **Cerda-Maira FA, Pearce MJ, Fuortes M, Bishai WR, Hubbard SR, Darwin KH.** 2010. Molecular analysis of the prokaryotic ubiquitin-like protein (Pup) conjugation pathway in *Mycobacterium tuberculosis*. Mol Microbiol **77:**1123-1135. http://dx.doi.org/10.1111/j.1365-2958.2010.07276.x.
25. **Rexer HU, Schaberle T, Wohlleben W, Engels A.** 2006. Investigation of the functional properties and regulation of three glutamine synthetase-like genes in *Streptomyces coelicolor* A3(2). Arch Microbiol **186:**447-458. http://dx.doi.org/10.1007/s00203-006-0159-8.
26. **Park JH, Cha CJ, Roe JH.** 2006. Identification of genes for mycothiol biosynthesis in *Streptomyces coelicolor* A3(2). J Microbiol **44:**121-125.
27. **Bursy J, Kuhlmann AU, Pittelkow M, Hartmann H, Jebbar M, Pierik AJ, Bremer E.** 2008. Synthesis and uptake of the compatible solutes ectoine and 5-hydroxyectoine by *Streptomyces coelicolor* A3(2) in response to salt and heat stresses. Appl Environ Microbiol **74:**7286-7296. http://dx.doi.org/10.1128/aem.00768-08.
28. **Oh SY, Shin JH, Roe JH.** 2007. Dual role of OhrR as a repressor and an activator in response to organic hydroperoxides in *Streptomyces coelicolor*. J Bacteriol **189:**6284-6292. http://dx.doi.org/10.1128/jb.00632-07.
29. **Horbal L, Fedorenko V, Bechthold A, Luzhetskyy A.** 2013. A transposon-based strategy to identify the regulatory gene network responsible for landomycin E biosynthesis. FEMS Microbiol Lett **342:**138-146. http://dx.doi.org/10.1111/1574-6968.12117.
30. **Derouaux A, Halici S, Nothaft H, Neutelings T, Moutzourelis G, Dusart J, Titgemeyer F, Rigali S.** 2004. Deletion of a cyclic AMP receptor protein homologue diminishes germination and affects morphological development of *Streptomyces coelicolor*. J Bacteriol **186:**1893-1897.
31. **Petzke L, Luzhetskyy A.** 2009. In vivo Tn5-based transposon mutagenesis of *Streptomycetes*. Appl Microbiol Biotechnol **83:**979-986. http://dx.doi.org/10.1007/s00253-009-2047-z.

32. **Ou X, Zhang B, Zhang L, Dong K, Liu C, Zhao G, Ding X.** 2008. SarA influences the sporulation and secondary metabolism in *Streptomyces coelicolor* M145. Acta Biochim Biophys Sin (Shanghai) **40:**877-882. http://dx.doi.org/10.1093/abbs/40.10.877.
33. **Uguru GC, Stephens KE, Stead JA, Towle JE, Baumberg S, McDowall KJ.** 2005. Transcriptional activation of the pathway-specific regulator of the actinorhodin biosynthetic genes in *Streptomyces coelicolor*. Mol Microbiol **58:**131-150. http://dx.doi.org/10.1111/j.1365-2958.2005.04817.x.
34. **Susstrunk U, Pidoux J, Taubert S, Ullmann A, Thompson CJ.** 1998. Pleiotropic effects of cAMP on germination, antibiotic biosynthesis and morphological development in *Streptomyces coelicolor*. Mol Microbiol **30:**33-46.
35. **Lu T, Zhu Y, Zhang P, Sheng D, Cao G, Pang X**. SCO5351 is a pleiotropic factor that impacts secondary metabolism and morphological development in *Streptomyces coelicolor*. FEMS microbiology letters. 2018;365(17). https://dx.doi.org/10.1093/femsle/fny150.
36. **Bishop A, Fielding S, Dyson P, Herron P.** 2004. Systematic insertional mutagenesis of a *streptomycete* genome: a link between osmoadaptation and antibiotic production. Genome Res **14:**893-900. http://dx.doi.org/10.1101/gr.1710304.
37. **Lu Y, He J, Zhu H, Yu Z, Wang R, Chen Y, Dang F, Zhang W, Yang S, Jiang W.** 2011. An orphan histidine kinase, OhkA, regulates both secondary metabolism and morphological differentiation in *Streptomyces coelicolor*. J Bacteriol **193:**3020-3032. http://dx.doi.org/10.1128/jb.00017-11.
38. **Song E, Rajesh T, Lee BR, Kim EJ, Jeon JM, Park SH, Park HY, Choi KY, Kim YG, Yang YH, Kim BG.** 2013. Deletion of an architectural unit, leucyl aminopeptidase (SCO2179), in *Streptomyces coelicolor* increases actinorhodin production and sporulation. Appl Microbiol Biotechnol **97:**6823-6833. http://dx.doi.org/10.1007/s00253-013-4847-4.
39. **Kang SH, Huang J, Lee HN, Hur YA, Cohen SN, Kim ES.** 2007. Interspecies DNA microarray analysis identifies WblA as a pleiotropic down-regulator of antibiotic biosynthesis in *Streptomyces*. J Bacteriol **189:**4315-4319. http://dx.doi.org/10.1128/jb.01789-06.
40. **Noh JH, Kim SH, Lee HN, Lee SY, Kim ES.** 2010. Isolation and genetic manipulation of the antibiotic down-regulatory gene, *wblA* ortholog for doxorubicin-producing *Streptomyces* strain improvement. Appl Microbiol Biotechnol **86:**1145-1153. http://dx.doi.org/10.1007/s00253-009-2391-z.
41. **Rabyk M, Ostash B, Rebets Y, Walker S, Fedorenko V.** 2011. S*treptomyces ghanaensis* pleiotropic regulatory gene *wblA*(gh) influences morphogenesis and moenomycin production. Biotechnol Lett **33:**2481-2486. http://dx.doi.org/10.1007/s10529-011-0728-z.
42. **Nah JH, Park SH, Yoon HM, Choi SS, Lee CH, Kim ES.** 2012. Identification and characterization of *wblA*-dependent *tmcT* regulation during tautomycetin biosynthesis in *Streptomyces sp*. CK4412. Biotechnol Adv **30:**202-209. http://dx.doi.org/10.1016/j.biotechadv.2011.05.004.
43. **Floriano B, Bibb M.** 1996. *afsR* is a pleiotropic but conditionally required regulatory gene for antibiotic production in *Streptomyces coelicolor* A3(2). Mol Microbiol **21:**385-396.

44. **Tanaka A, Takano Y, Ohnishi Y, Horinouchi S.** 2007. AfsR recruits RNA polymerase to the *afsS* promoter: a model for transcriptional activation by SARPs. J Mol Biol **369:**322-333. http://dx.doi.org/10.1016/j.jmb.2007.02.096.
45. **Mingyar E, Sevcikova B, Rezuchova B, Homerova D, Novakova R, Kormanec J.** 2014. The sigma(F)-specific anti-sigma factor RsfA is one of the protein kinases that phosphorylates the pleiotropic anti-anti-sigma factor BldG in *Streptomyces coelicolor* A3(2). Gene **538:**280-287. http://dx.doi.org/10.1016/j.gene.2014.01.041.
46. **Kim ES, Song JY, Kim DW, Chater KF, Lee KJ.** 2008. A possible extended family of regulators of sigma factor activity in *Streptomyces coelicolor*. J Bacteriol **190:**7559-7566. http://dx.doi.org/10.1128/jb.00470-08.
47. **Li W, Ying X, Guo Y, Yu Z, Zhou X, Deng Z, Kieser H, Chater KF, Tao M.** 2006. Identification of a gene negatively affecting antibiotic production and morphological differentiation in *Streptomyces coelicolor* A3(2). J Bacteriol **188:**8368-8375. http://dx.doi.org/10.1128/jb.00933-06.
48. **Sumby P, Smith MC.** 2002. Genetics of the phage growth limitation (Pgl) system of *Streptomyces coelicolor* A3(2). Mol Microbiol **44:**489-500. http://dx.doi.org/10.1046/j.1365-2958.2002.02896.x.
49. **Yu TW, Hopwood DA.** 1995. Ectopic expression of the *Streptomyces coelicolor whiE* genes for polyketide spore pigment synthesis and their interaction with the *act* genes for actinorhodin biosynthesis. Microbiology **141 (Pt 11):**2779-2791. http://dx.doi.org/10.1099/13500872-141-11-2779.
50. **Alvarez MA, Fu H, Khosla C, Hopwood DA, Bailey JE.** 1996. Engineered biosynthesis of novel polyketides: properties of the *whiE* aromatase/cyclase. Nat Biotechnol **14:**335-338. http://dx.doi.org/10.1038/nbt0396-335.
51. **Domenech P, Reed MB, Barry CE, 3rd.** 2005. Contribution of the *Mycobacterium tuberculosis* MmpL protein family to virulence and drug resistance. Infect Immun **73:**3492-3501. http://dx.doi.org/10.1128/iai.73.6.3492-3501.2005.
52. **Bordo D, Bork P.** 2002. The rhodanese/Cdc25 phosphatase superfamily. Sequence-structure-function relations. EMBO Rep **3:**741-746. http://dx.doi.org/10.1093/embo-reports/kvf150.
53. **Diaz M, Esteban A, Fernandez-Abalos JM, Santamaria RI.** 2005. The high-affinity phosphate-binding protein PstS is accumulated under high fructose concentrations and mutation of the corresponding gene affects differentiation in *Streptomyces lividans*. Microbiology **151:**2583-2592. http://dx.doi.org/10.1099/mic.0.27983-0.
